# Parental perceptions of risky play and preschooler’s motor creativity: mastery motivation as mediator and gender as moderator

**DOI:** 10.3389/fpsyg.2026.1856120

**Published:** 2026-05-29

**Authors:** Nezahat Hamiden Karaca, Ali İbrahim Can Gözüm, Ümit Ünsal Kaya, Halil Uzun, Şermin Metin

**Affiliations:** 1 Department of Early Childhood Education, Faculty of Education, Afyon Kocatepe University, Afyonkarahisar, Türkiye; 2 Department of Early Childhood Education, Dede Korkut Faculty of Education, Kafkas University, Kars, Türkiye; 3 School of Foreign Languages, Afyon Kocatepe University, Afyonkarahisar, Türkiye; 4 Department of Child Development, Faculty of Health Sciences, Tarsus University, Mersin, Türkiye; 5 Department of Family Sciences, School of Public Health, University of Maryland, College Park, MD, United States; 6 Department of Early Childhood Education, Faculty of Education, Hasan Kalyoncu University, Gaziantep, Türkiye

**Keywords:** gender, motivation, motor creativity, preschoolers, risky play

## Abstract

**Introduction:**

Risky play, characterized by activities involving uncertainty, excitement, and moderate physical risk, is essential for children’s cognitive, emotional, and motor development. However, the precise pathways through which it impacts motor creativity, along with the underlying psychological mechanisms, remain insufficiently explored. This study investigates the direct relationship between parental perceptions of risky play and preschoolers’ motor creativity, examining the mediating role of mastery motivation and the moderating effect of gender.

**Methods:**

Data were collected from 367 typically developing preschoolers (aged 48–72 months) across public preschools in Türkiye. The proposed moderated mediation framework was evaluated using path analysis within Structural Equation Modeling (SEM).

**Results:**

Structural analysis demonstrates that children engaging in higher levels of risky play exhibit significantly greater motor creativity, confirming that physically and cognitively stimulating environments promote adaptive movement patterns and creative problem-solving. Furthermore, mastery motivation functions as a significant mediator, indicating that the sense of challenge and persistence inherent in risky play enhances children’s intrinsic drive to explore and refine motor skills, thereby boosting creative expression. Significant gender moderation was also observed, with boys displaying stronger associations between risky play, mastery motivation, and motor creativity compared to girls.

**Discussion:**

The study underscores that risky play actively fosters early childhood motor creativity and embodied problem-solving. Crucially, the findings highlight that parental perceptions serve as vital environmental gatekeepers, shaping play contexts that can either constrain or dynamically support child autonomy, robust exploration, and safe risk-taking.

## 1 Introduction

Motor creativity, defined as the ability to generate new and adaptable movement patterns in response to environmental and situational demands, is a critical component of children’s physical and cognitive development (Olivares-Chavarro, 2014; Oppici et al., 2020). Research has shown that motor creativity enhances problem-solving skills, coordination, and cognitive flexibility, making it a key factor in learning and adaptation during early childhood (Orth et al., 2017; Runco and Acar, 2012). Given its importance, it is essential to identify the factors that promote motor creativity in early childhood. One potential contributor is risky play, which involves physically and cognitively challenging activities that require children to navigate uncertainty, explore new movement possibilities, and develop creative solutions to environmental challenges (Sandseter and Kleppe, 2019). However, opportunities to engage in risky play are shaped by parental attitudes, which can either encourage or restrict children’s participation in physically demanding activities. Studies indicate that while many parents acknowledge the developmental benefits of risky play, they also express concerns about potential injuries, social judgments, and safety regulations (Brussoni et al., 2012; Cevher-Kalburan and Ivrendi, 2016; Giles et al., 2018; Gull et al., 2018; Jelleyman et al., 2019; Little and Eager, 2010; Little et al., 2012). Some parents support children’s involvement by offering encouragement and supervision, whereas others impose strict limitations out of fear of possible consequences (Allin et al., 2014; Ball et al., 2012). When excessive, such restrictions may limit children’s ability to explore diverse movement experiences and potentially constrain their motor creativity. Historically, traditional plays and games have served as the first and most natural environments stimulating children’s sensorimotor development (Pellegrini and Smith, 1998). In parallel, commercially designed educative tools, such as Early Learning Center toys and other specially designed manipulatives, have been developed to support and enrich children’s naturally occurring developmental processes (Hirsh-Pasek et al., 2009).

Beyond parental perceptions of risky play, motivation is another important factor influencing children’s engagement in play and creative movement. Risky play inherently offers opportunities for children to test their abilities, overcome challenges, and gain a sense of accomplishment, all of which are core drivers of motivation (Gilmore, 2018). Research demonstrates that when children succeed in managing physical challenges, they develop greater confidence in their abilities, which in turn strengthens their motivation to engage in similar activities in the future (Gilmore and Cuskelly, 2025; Çelik et al., 2024; Gilmore, 2018; Wang et al., 2011). This heightened motivation may enhance motor creativity, as motivated children are more likely to experiment with novel movement solutions and continue to develop adaptive motor skills (Beghetto and Kaufman, 2016). Despite these theoretical connections, empirical studies exploring the mediating role of motivation in the relationship between risky play and motor creativity remain limited.

Another critical factor shaping these relationships is gender. Research consistently shows that boys and girls engage in risky play at various levels. Boys are more likely to participate in physically intense and high-risk activities, whereas girls tend to engage in more structured and cooperative forms of play (Little and Wyver, 2008; Play Wales, 2023; Sandseter and Kennair, 2011). Consequently, boys may have greater opportunities to foster motor creativity through a broader repertoire of movement experiences, while girls may encounter fewer opportunities to benefit from risky play (Brussoni et al., 2015). Moreover, gender differences in motivation may further influence the extent to which risky play contributes to motor creativity. Boys, who are often more strongly encouraged to persist in physically demanding tasks (Gleave, 2008), may derive greater motivational benefits from risky play, which in turn enhances their motor creativity. Understanding how gender moderates these relationships is essential for designing inclusive play environments that support the development of creative movement skills in both boys and girls.

Despite the growing interest in the developmental benefits of motor creativity, a comprehensive framework that explains its antecedents and the factors influencing its development is still lacking. To address this gap, the present study investigates (1) the direct relationship between parental perceptions of risky play and motor creativity, (2) the mediating role of motivation, and (3) the moderating effect of gender. By integrating these factors into a unified model, this study aims to provide new insights into the mechanisms underlying motor creativity in early childhood and to offer practical implications for educators and parents.

### 1.1 Conceptual framework and hypotheses development

#### 1.1.1 Motor creativity

Motor creativity refers to the capacity to generate new, flexible, and adaptive movement solutions in response to environmental and situational challenges (Olivares-Chavarro, 2014; Oppici et al., 2020). Unlike routine or repetitive movements, motor creativity involves exploratory movement patterns that foster children’s ability to solve physical problems, coordinate actions, and adapt to changing contexts (Runco, 2007; Orth et al., 2017). From a developmental perspective, motor creativity is not only an expression of motor skill competence but also reflects cognitive flexibility and embodied problem-solving, enabling children to interact with their environment in innovative ways (Richard et al., 2018; Wyrick, 1968).

The preschool years represent a critical stage when children most intensively display creativity and rapidly develop their motor skills. During this period, motor creativity provides more than just variation in bodily movements; it also supports social–emotional development, problem-solving abilities, and independence in action (Lorenzo-Lasa et al., 2007; Wang, 2003). Through play and movement, children learn to cooperate with peers and take risks in trying innovative approaches (Elkind, 2007; Little and Wyver, 2008). Research highlights that environmental arrangements, family characteristics, and teacher attitudes are decisive in shaping the development of motor creativity. For example, maternal age and occupation have been shown to predict children’s fluency and originality scores (Karaca and Aral, 2017), environments that permit risky play enhance motor creativity (Sandseter and Kennair, 2011), and movement-based activities such as sports and music positively influence motor creativity scores (Cheung, 2010; Pamuk et al., 2022). In this sense, fostering motor creativity requires a holistic approach that nourishes not only physical but also cognitive, social, and emotional development.

Motor creativity can be conceptualized as a multidimensional construct encompassing fluency, originality, and flexibility, with imagination functioning as an integrative component of creative expression (Torrance, 1981; Wyrick, 1968; Runco, 2007). From an embodied cognition perspective, these dimensions emerge through the dynamic coupling of perception, action, and cognition (Gibson, 2014; Thelen and Smith, 1994). Consistent with Dynamic Systems Theory, motor creativity is not a fixed trait but a self-organizing capacity shaped by ongoing interactions among the individual, task, and environment (Thelen, 2000; Smith and Thelen, 2003). These interactions are further influenced by sensorimotor experiences, peer engagement, and environmental affordances, which collectively shape developmental trajectories (Pellegrini and Smith, 1998; Gibson, 2014). In early childhood, flexibility and imagination become particularly salient, as children engage in embodied exploration and improvisational play, relying heavily on action-based and non-verbal forms of meaning-making prior to the full maturation of abstract symbolic reasoning (Runco, 2007; Pellegrini and Smith, 1998; Richard et al., 2018).

#### 1.1.2 Risky play

Play, defined as a voluntary activity that emerges spontaneously through social interaction and exploration (Frost, 2010), takes a specific form in risky play, which is generally characterized as thrilling and exciting play involving a risk of physical injury (Sandseter, 2009a, 2009b). By its very nature, play emphasizes uncertainty, novelty, and flexibility, with a focus on the process rather than the outcome (Caillois, 1962; Lester and Russell, 2008). Within this process, unexpected risky situations may naturally arise. In fact, most individuals encounter risk elements in their daily lives. The key lies not in eliminating such risks but in preparing to manage them through experience and learning (Küçük, 2010).

Early childhood is particularly important for acquiring these experiences, as children tend to prefer challenging games that involve risk from a young age (Boyer, 2006; Sandseter, 2010; Stephenson, 2003) and are believed to possess an innate inclination toward risky play (Brussoni et al., 2012). Early experiences play a critical role in development (Sweatman and Warner, 2009), and numerous studies have demonstrated that engaging in both play and risky play during early childhood contributes to multiple developmental domains (Greenfield, 2004; Sandseter, 2007, 2009a, 2009b; Sandseter, 2010; Stephenson, 2003; Waters and Begley, 2007). Moreover, while risky play provides enjoyable experiences, it also inadvertently fulfills educational functions (Spinka et al., 2001).

In modern Western societies, however, children’s safety has become a central focus across nearly all contexts, including play. Excessive emphasis on safety within children’s play environments has emerged as a concern. Although avoiding injury is important, children require exposure to challenges and diverse stimuli to achieve typical physical and psychological development (Sandseter, 2012). For instance, children attending forest schools regularly engage in risk-taking and risky play, whereas those in more traditional schools encounter fewer such opportunities due to institutional constraints. Studies report that children spend much of their free time in structured and institutionalized settings (Meire, 2013; Valentine and McKendrick, 1997), have limited opportunities for outdoor play, and increasingly lead sedentary lifestyles (Louv, 2008).

Research further suggests that the concept of risk is socially constructed and varies both within and across cultures (Little and Eager, 2010). While adults tend to encourage mental risk-taking in children, they often perceive physical risk as negative and disallow it (Little and Wyver, 2008). Concerns about safety, anxiety, fear, and overprotective parenting attitudes have spurred growing interest in how opportunities for risky play are regulated (Adams, 2001; Apter, 2007; Baum and McMurray-Schwarz, 2004; Brussoni et al., 2012; Brussoni et al., 2015; Brussoni et al., 2017; Brussoni et al., 2018; Carver et al., 2008; Cevher Kalburan, 2014; Cevher-Kalburan and Ivrendi, 2016; Christensen, 2002; Coe, 2017; Furedi, 2001; Güler and Demir, 2016; Harper, 2017; Kleppe, 2018; Lester and Russell, 2008; Little, 2006, 2009; Little and Wyver, 2008; Little et al., 2011; Little et al., 2012; Moralı, 2019; McFarland and Laird, 2018; Morrongiello and Lasenby-Lessard, 2007; Nikiforidou, 2017; Play England, 2011; Sandseter, 2014; Ünüvar and Kanyılmaz, 2017). Conversely, some scholars argue that shielding children from even the smallest dangers while ignoring the benefits of risky play may create greater long-term harm (Skenazy, 2009). Depriving children of age-appropriate risky play may hinder normal development (Alexander et al., 2014).

Over the past decade, opportunities for outdoor and risky play have significantly declined across Europe (Clements, 2004). Although reducing or eliminating risky play may yield short-term benefits, in the long term such restrictions may foster inactivity, low self-confidence (Little and Wyver, 2008), obesity, mental health problems, diminished independence, and weaker learning, perception, and judgment skills (Eager and Little, 2011). Children who do not engage in risky play are also more likely to encounter fitness and motor skill difficulties in adolescence compared to peers who do (Kantomaa et al., 2011). Importantly, parental practices play a critical role in shaping children’s ability to develop independent risk assessment skills (Little, 2010). Safety concerns often lead adults to impose restrictions on risk-taking in play, depriving children of fundamental needs such as running, playing outdoors in the snow, or exploring restricted areas. This perspective highlights that risk should not be viewed solely as a danger to be avoided, but rather as something to be managed—acknowledging both its potential benefits and hazards (Ball et al., 2012; Little, 2010).

### 1.2 Motor creativity and its relationship with parental perceptions of risky play

Risky play is characterized by children’s engagement in activities that involve uncertainty, excitement, and a moderate degree of physical risk, such as climbing, balancing on unstable surfaces, jumping from heights, and rough-and-tumble play (Sandseter, 2009a, 2009b). These activities are considered essential for children’s physical, cognitive, and socio-emotional development because they provide opportunities for problem-solving, risk assessment, and adaptive motor learning (Brussoni et al., 2015; Stephenson, 2003). By encountering physically challenging and unpredictable situations, children strengthen their motor skills, explore movement variability, and develop self-confidence (Dereobalı and Çandır, 2021; Moyles, 2012; Sandseter and Kleppe, 2019). Through play as a natural part of development, children build exploration, experimentation, and risk-taking abilities, and outdoor risky play in particular enriches their experiences, pushes them beyond the ordinary, and facilitates innovative thinking (Greenfield, 2004; Tovey, 2010). In this respect, unstructured and dynamic play environments offer an ideal context for fostering motor creativity (Diamond, 2000; Vygotsky, 1981). Specifically, risky play has been identified as a significant contributor to motor creativity because it involves movement variability, adaptability, and real-time problem-solving in unpredictable contexts (Boyer, 2006; Karaca et al., 2024; Stephenson, 2003). By presenting open-ended movement challenges and promoting creativity through spontaneous and improvisational actions, risky play requires children to assess risks, regulate movements, and adjust their actions based on environmental feedback—all of which enhance motor creativity (Sandseter and Kennair, 2011).

Despite these documented benefits, risky play is often restricted due to safety concerns, parental anxieties, and societal perceptions regarding acceptable risk-taking in childhood. These constraints may inadvertently limit children’s opportunities to develop motor creativity (Akdemir et al., 2023; Ball et al., 2012; Brussoni et al., 2012; Cevher-Kalburan and Ivrendi, 2016; Gleave, 2008; Gull et al., 2018; Little, 2015; Little and Eager, 2010; Little and Wyver, 2008; McFarland and Laird, 2018; Sandseter, 2009a, 2009b; Storli and Hansen Sandseter, 2019). In particular, parents’ perceptions and attitudes toward risky play exert a strong influence on children’s participation. Research has noted inconsistencies between parents’ recognition of the advantages of risky play and their willingness to allow children to engage in it (Cevher-Kalburan and Ivrendi, 2016; Creighton et al., 2017; Jelleyman et al., 2019; Little, 2010, 2015; Niehues et al., 2013, 2015). Furthermore, studies have identified differences between mothers and fathers, with some findings suggesting that fathers are generally more tolerant of risky play than mothers (Brussoni et al., 2013; Brussoni and Olsen, 2013; Creighton et al., 2017; Little, 2010, 2015; Niehues et al., 2013, 2015). Nonetheless, Brussoni et al. (2021) argue that intervention programs targeting mothers can increase their tolerance for risky play. While parents often remain cautious, Sandseter (2009a, 2009b) emphasizes the growing recognition that children both desire and need engagement in risky play.

Building on this theoretical and empirical foundation, the present study proposes the following hypothesis:

*H_1_*: Parental perceptions of risky play have a direct positive effect on children’s motor creativity.

#### 1.2.1 The role of motivation

Motivation is a key psychological mechanism that drives individuals to initiate, sustain, and persist in activities (Ryan and Deci, 2000; Vasilopoulos and Dumontheil, 2024). In childhood, motivation plays a critical role in learning, exploration, and skill development, shaping children’s willingness to engage in challenging activities and experiment with new movement patterns (Carlton and Winsler, 1998). The motivational drive also fosters motor creativity by encouraging children to explore a variety of movement solutions (Murayama and Elliot, 2009; Topçu, 2015). Fung and Chung (2022) highlight that children’s intense excitement during imaginative and fantasy play can lead to heightened cognitive arousal, which in turn contributes to manipulating ideas and generating novel ones.

Although previous studies have examined the relationship between motivation and creativity (Beghetto and Kaufman, 2016; Runco and Acar, 2012), there is limited research exploring the specific role of motivation in the link between risky play and motor creativity. Scholars suggest that an indirect relationship exists between risky play and motivation. Gilmore (2018) notes that when children are provided with opportunities to test their abilities in unpredictable and dynamic environments, their sense of competence and persistence is strengthened. Empirical studies further indicate that positive experiences in risky play contribute to increased motivation for ongoing participation in physically challenging activities (Gilmore and Cuskelly, 2025; Wang et al., 2011). Children who engage in risky play and play in risky environments have been found to be more creative, more self-confident, and less likely to exhibit signs of stress (Brussoni et al., 2017; Gull et al., 2018). Such experiences also help them develop persistence and motivation to overcome challenges and achieve goals (McFarland and Laird, 2018; Sandseter, 2009a; Tee, 2022).

Thus, motor creativity may not be solely a direct outcome of risky play, but rather partly explained by motivational processes arising from participation in challenging and uncertain play experiences. Building on these theoretical and empirical insights, the following hypotheses are proposed:

*H_2_*: Parental perceptions of risky play have a direct positive effect on children’s motivation.

*H_3_*: Children’s motivation has a direct positive effect on their motor creativity.

*H_3a_*: Children’s motivation significantly mediates the relationship between parental perceptions of risky play and children’s motor creativity.

#### 1.2.2 The role of gender

Gender is considered an important factor in shaping children’s participation in risky play, their motivation, and ultimately their motor creativity (Sandseter and Kennair, 2011). Research has consistently shown that boys are more likely than girls to engage in physically intense and high-risk activities, which can lead to differences in motor skill development and creative movement patterns (Little and Wyver, 2008; Play Wales, 2023). Socialization processes play a central role in shaping these gender differences, with scholars emphasizing the influence of adults’ gendered attitudes (Brussoni et al., 2015; Gleave, 2008; Little, 2015).

In addition to socialization, gender-based differences in motivation may further affect the relationship between risky play and motor creativity. Motivation, defined as a child’s persistent effort to overcome challenges and develop competence (Morgan et al., 1990), has been shown to play a crucial role in creative movement exploration (Saracho, 2019). Since boys are often encouraged to persist in physically demanding tasks, they may experience stronger motivational drives in response to risky play (Gleave, 2008). Boys’ greater likelihood of participating in physically challenging play and experiencing mastery in such contexts may place them in a better position to develop creative movement solutions (Karaca et al., 2024). By contrast, girls may require additional support or alternative play opportunities to foster motor creativity to a similar degree. Considering these factors, gender is expected to moderate the relationship between risky play, motivation, and motor creativity. Accordingly, the following hypotheses are proposed:

*H_4a_*: Gender moderates the relationship between parental perceptions of risky play and motivation, such that boys score higher than girls.

*H_4b_*: Gender moderates the relationship between parental perceptions of risky play and motor creativity, such that boys score higher than girls.

*H_4c_*: Gender moderates the relationship between motivation and motor creativity, such that boys score higher than girls.

#### 1.2.3 Study context and contribution

This study aimed to examine the direct and indirect relationships among motor creativity, parental perceptions of risky play, motivation, and gender in children aged 4–6 years. Research on motor creativity, a domain that significantly contributes to preschool children’s development has been steadily increasing. In recent years, particular emphasis has been placed on studies that support the development of motor creativity through interventions and activities. For instance, motor creativity has been linked to sports and physical activity (Coppola et al., 2024; Karaca and Aral, 2017; Khorkova et al., 2024; Pamuk et al., 2022; Richard et al., 2018; Tocci et al., 2022; Wang, 2003), music education and dance (Thomaidou et al., 2021; Trecroci et al., 2023; Şenol and Karaca, 2025), play and social participation (Ourda et al., 2025), and dramatic play (Panagiotaki et al., 2023). Correlational studies have also examined motor creativity in relation to other domains, such as creativity (Scibinetti et al., 2011), motor skills (Sturza-Milić, 2012), peer play behaviors (Karaca et al., 2020), scientific process skills (Can Yaşar et al., 2022), general play (Sherrill et al., 1977), motor competence (Centelles, 2020), self-perception (Bournelli et al., 2009), the impact of physical activity and screen time (Ghanamah, 2025), aesthetic and artistic psychology (Vasilopoulos and Dumontheil, 2024), and demographic variables (Karaca et al., 2020).

However, to better support the development of motor creativity and to design effective programs for children, it is necessary to clarify the domains with which motor creativity is associated. While previous studies have separately examined the contributions of risky play, motivation, and creativity to motor creativity, it remains important to investigate how these variables interact within a single framework. By conceptualizing motivation as a mediator and gender as a moderator, this study seeks to provide a more nuanced understanding of the mechanisms linking parental perceptions of risky play to motor creativity. In line with the literature, we hypothesized that motor creativity and parental perceptions of risky play would be significantly associated, and that motivation and gender would significantly mediate and moderate these associations.

This figure illustrates the conceptual framework of the proposed model, highlighting key constructs and hypothesized relationships.

## 2 Methods

### 2.1 Research model

**Figure 1:**
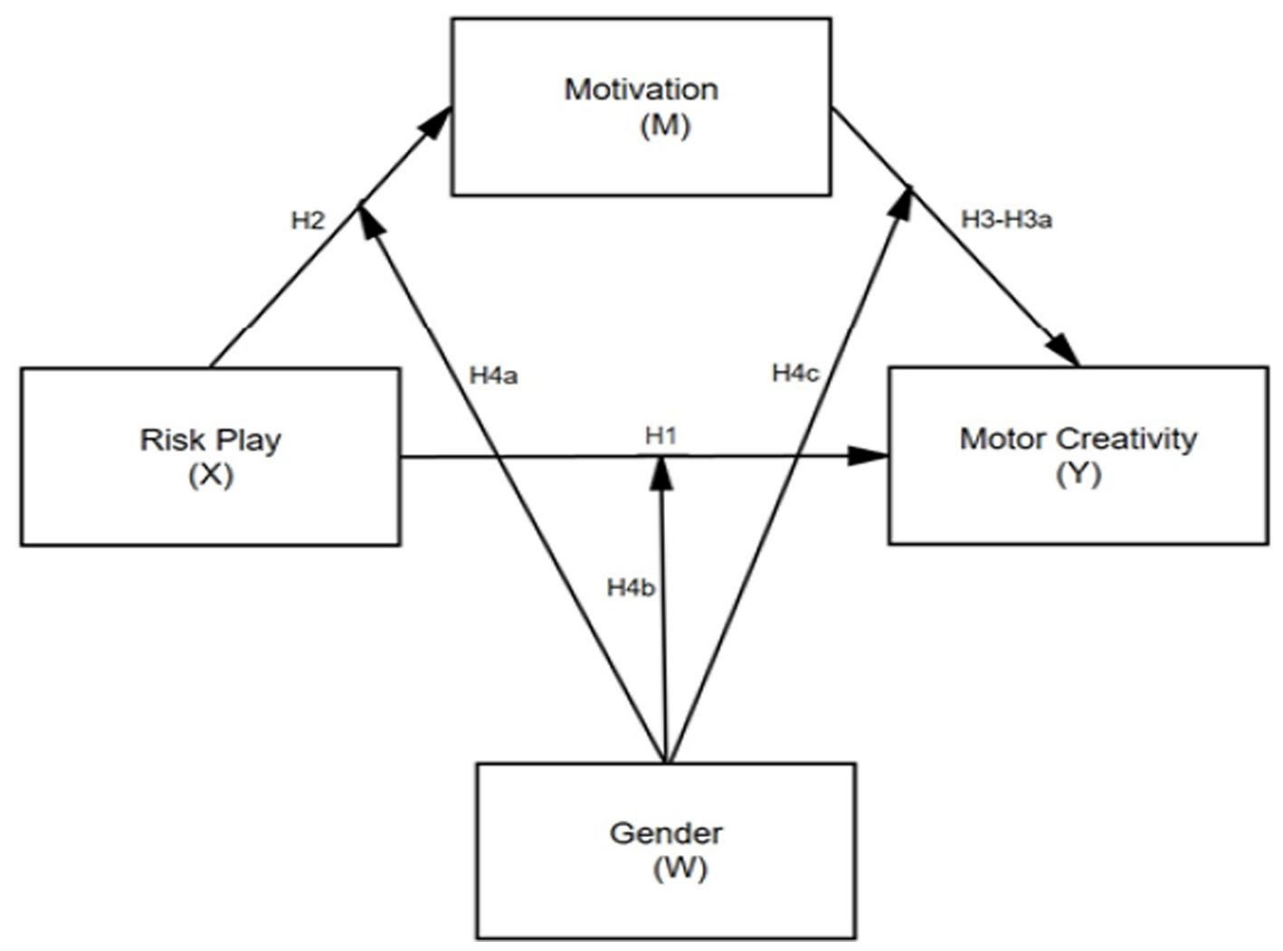
Theoretical representation of the proposed model.

Based on the theoretical framework and previous research findings, this study proposes a conceptual model that examines the relationships among parental perception toward risky play, motivation, motor creativity, and gender as a moderator. Figure 1 illustrates the proposed research model, which integrates these hypotheses and highlights the expected direct, mediating, and moderating relationships.

### 2.2 Study design

This study adopts a correlational and explanatory research design to examine the relationships among parental perception toward risky play, mastery motivation, and motor creativity, while also considering children’s gender as a moderating variable. To analyze these relationships, the study employs path analysis, a statistical approach that enables the simultaneous testing of direct, indirect, and moderated associations within a unified framework (Kline, 2016; Schumacker and Lomax, 2004). Path analysis is particularly suitable for examining mediation and moderation effects using observed variables, making it an appropriate method for testing the hypothesized pathways between parental perception toward risky play, mastery motivation, and motor creativity.

Path analysis examined the direct and indirect effects of parental perceptions of risky play on children’s motor creativity, testing mastery motivation as a mediator and gender as a moderator to elucidate the underlying mechanisms.

### 2.3 Participants

The participants of this study consisted of 367 preschool children aged 4 to 6 years, who were enrolled in public preschools in Afyonkarahisar, Türkiye. To ensure a representative and diverse sample, a two-stage cluster random sampling method was employed (Creswell and Creswell, 2018; Tabachnick and Fidell, 2013).

In the first stage, 10 public preschools were randomly selected from the list of preschools provided by the Afyonkarahisar Provincial Directorate of National Education. These schools were chosen to represent a balanced distribution of socioeconomic backgrounds, ensuring variability in children’s play experiences and developmental environments. The inclusion of multiple schools helped to increase external validity by minimizing the influence of school-specific factors (Fraenkel and Wallen, 2009). In the second stage, a random selection of children was conducted from within the selected schools. A total of 367 children were randomly chosen based on school records, ensuring that the sample reflected the overall preschool population in the region. Only children whose parents provided written informed consent were included in the study. Children with developmental delays or diagnosed disabilities were excluded to maintain sample homogeneity and focus on typically developing children. This sampling strategy enhances the generalizability of the findings, as cluster random sampling is particularly useful in educational research when individual random selection is impractical (Teddlie and Yu, 2007). By incorporating multiple schools and a diverse socioeconomic background, the study aimed to capture a more comprehensive picture of the relationships between risky play, motivation, and motor creativity. A detailed breakdown of the participants’ demographic characteristics is presented in Table 1.

**Table 1:** Demographic characteristics of the participants (*N* = 367).

Variable	Category	*n*	%
Child’s age (years)	4.0–4.4	65	17.7
	4.5–4.9	72	19.6
	4.10–5.1	76	20.7
	5.2–5.6	79	21.5
	5.7–6.0	75	20.4
Gender	Female	182	49.6
	Male	185	50.4
Birth order	Firstborn	196	53.4
	Middle	79	21.5
	Youngest	92	25.1
Number of children in family	One child	79	21.5
	More than one	288	78.5
Previous preschool education	Yes	210	57.2
	No	157	42.8
Mother’s age	Below 29	96	26.2
	30–39	234	63.8
	40 and above	37	10.1
Father’s age	Below 29	29	7.9
	30–39	257	70.0
	40 and above	81	22.1
Mother’s education	Primary and middle school	137	37.3
	High school	76	20.7
	University	154	42.0
Father’s education	Primary and middle school	81	22.1
	High school	108	29.4
	University	178	48.5
Mother’s occupation	Homemaker	212	57.8
	Government employee	107	29.2
	Worker	26	7.1
	Self-employed	22	6.0
Father’s occupation	Government employee	113	30.8
	Worker	151	41.1
	Self-employed	103	28.1

### 2.4 Measurement tools

#### 2.4.1 Demographic information form

A Demographic Information Form was developed by researchers to gather information about the children and their families. This form included the child’s age, gender, birth order, number of siblings, previous preschool education experience, parental age, parental education level, and parental occupation. The data were obtained from school records after obtaining parental consent, ensuring accuracy and reliability.

#### 2.4.2 The scale for the perceptions towards risky play at early childhood—parent form (SATRPEC-PF)

Parental perceptions of risky play were measured using the *Scale for the Perceptions Towards Risky Play at Early Childhood—Parent Form* (SATRPEC-PF) (Karaca, 2020). This 28-item Likert-type scale consists of five subdimensions: *Beliefs, Distinguishing Risky Behaviors, Supporting Children, Experiencing Anxiety,* and *Parental Support*. Participants rated each item on a 5-point scale (1 = Strongly Disagree, 5 = Strongly Agree). Overall Cronbach’s alpha reliability coefficient for the scale was 0.919, indicating high internal consistency. Confirmatory factor analysis (CFA) confirmed the five-factor structure and demonstrated good model fit. In the present study, the scale was completed by 257 mothers, yielding a Cronbach’s alpha reliability coefficient of 0.83.

#### 2.4.3 Dimensions of mastery questionnaire (DMQ18)

Children’s motivation was assessed using the *Dimensions of Mastery Questionnaire* (DMQ18). Originally developed by Morgan et al. (2015) and revised over time, this instrument evaluates mastery motivation across seven dimensions: *cognitive persistence, gross motor persistence, social persistence with adults, social persistence with children, mastery pleasure, negative reactions,* and *general competence*. The preschool version consists of 39 items rated on a 5-point Likert scale (1 = Not at all like the child, 5 = Exactly like the child). The scale is completed by teachers who regularly observe the child in educational contexts. The Turkish adaptation was conducted by Özbey and Dağlıoğlu (2017), demonstrating satisfactory reliability (*α* = 0.77 to 0.90) and strong test–retest reliability (r = 0.85).

#### 2.4.4 Thinking creatively in action and movement test (TCAM)

Motor creativity was measured using the *Thinking Creatively in Action and Movement Test* (TCAM) (Torrance, 1981), designed for children aged 3 to 8 years. The test assesses three dimensions of creativity: *fluency, originality,* and *imagination*. It comprises four activities: the first, third, and fourth activities measure fluency and originality, while the second activity evaluates imagination. Each activity is scored according to standardized criteria. The second activity is rated during implementation on a five-point scale (1 = No action, 5 = Excellent), whereas the other three activities are scored post-implementation by trained experts. The Turkish adaptation was developed by Karaca and Aral (2017), ensuring both linguistic and cultural appropriateness for preschool-aged children in Türkiye. Reliability analyses revealed a test–retest reliability coefficient of 0.84, and validity studies confirmed strong consistency with comparable instruments.

### 2.5 Data collection

Prior to data collection, ethical approval was obtained from the Social and Human Sciences Research and Publication Ethics Committee of Afyon Kocatepe University. Written informed consent was also obtained from all parents through a *Parental Consent Form*, which detailed the study’s purpose, voluntary participation, and confidentiality assurances. Only children whose parents provided informed consent were included.

Data collection was conducted during the spring semester of the 2023–2024 academic year in preschools located in Afyonkarahisar, Türkiye. All assessments were administered in quiet, designated rooms within the schools to ensure that children were comfortable and free from distractions. School administrators and teachers facilitated scheduling and logistics.

Parents of participating children (*N* = 257) completed the SATRPEC-PF at home and returned the forms via school administration. Children’s motivation was evaluated by preschool teachers using the DMQ18, a process that took approximately 10–15 min per child. Motor creativity was individually assessed using the TCAM, administered by trained early childhood researchers in one-on-one sessions lasting 45–60 min. Throughout the assessment, researchers ensured that children remained engaged and comfortable. All data were anonymized and securely stored in line with ethical standards for research with young children. The study adhered strictly to ethical protocols to protect the rights and well-being of participants.

### 2.6 Data analysis

A series of statistical analyses were conducted to examine the hypothesized relationships among parental perceptions of risky play, mastery motivation, motor creativity, and gender. Prior to testing the research model, data screening and assumption testing were performed to ensure the accuracy, reliability, and appropriateness of the dataset for subsequent analyses (Tabachnick and Fidell, 2013).

### 2.7 Assumption testing

To verify that the dataset satisfied the statistical assumptions required for path analysis, a series of diagnostic procedures were undertaken. Since all variables in the model (parental perceptions of risky play, mastery motivation, motor creativity, and gender) were treated as observed variables, their distributional characteristics and statistical properties were carefully examined.

First, preliminary screening was carried out to identify missing values and extreme outliers. Cases with substantial missing data or values that substantially deviated from the normal distribution were excluded from further analyses. Univariate normality was assessed by examining skewness and kurtosis values, which for all variables (see Table 2) fell within the acceptable threshold of ±3. In addition, Mardia’s test indicated a multivariate kurtosis value of 2.47, supporting the assumption of multivariate normality (Kline, 2016).

**Table 2:** Correlations among constructs, means, standard deviations, skewness, and kurtosis.

Pearson’s correlation coefficients					Descriptive stats		Normality stats	
Variables		Risky play	Motivation	Motor creativity	M	SD	Skewness	Kurtosis
Whole sample *N* = 367	Risky play	1			77.24	9.51	1.69	2.85
	Motivation	0.736^**^	1		88.72	14.47	2.03	2.65
	Motor creativity	0.790^**^	0.775^**^	1	261.26	31.87	0.457	1.86
Girls *N* = 182	Risky play	1			76.48	9.68	1.62	2.52
	Motivation	0.719^**^	1		87.58	14.98	2.10	2.35
	Motor creativity	0.715^**^	0.772^**^	1	257.54	32.10	0.545	1.72
Boys *N* = 185	Risky play	1			78.01	9.30	1.79	2.44
	Motivation	0.753^**^	1		89.87	13.89	1.96	2.46
	Motor creativity	0.774^**^	0.785^**^	1	265.05	31.29	0.370	1.45

**, Correlation is significant at the 0.01 level (2-tailed). *, Correlation is significant at the 0.05 level (2-tailed).

To assess multicollinearity, variance inflation factor (VIF) values (1.12–1.36) and tolerance values (0.73–0.89) were examined, indicating no multicollinearity concerns. Linearity and homoscedasticity assumptions were also met, as confirmed by scatterplots and standardized residual analyses, with residuals evenly distributed around zero (Schumacker and Lomax, 2004).

### 2.8 Path analysis

Path analysis was employed to test the direct, indirect, and moderating associations hypothesized in the conceptual model. Specifically, the analysis examined the direct relationship between parental perceptions of risky play and motor creativity (H_1_), as well as the association between parental perceptions of risky play and mastery motivation (H_2_).

Mediation effects were tested using the bootstrap method with 5,000 resamples and 95% confidence intervals to determine whether mastery motivation mediated the relationship between parental perceptions of risky play and motor creativity (H_3a_) (Hayes, 2018). Moderation effects were assessed to examine whether gender influenced the associations between parental perceptions of risky play and mastery motivation (H_4a_), between parental perceptions of risky play and motor creativity (H_4b_), and between mastery motivation and motor creativity (H4c). Multi-group path analysis was conducted for moderation testing, and statistical significance was determined using the critical ratio (Z-score), with values exceeding 1.96 considered significant (Baron and Kenny, 1986; Frazier et al., 2004).

### 2.9 Model fit evaluation

The adequacy of the proposed model was evaluated using multiple fit indices widely recommended in structural equation modeling. The chi-square to degrees of freedom ratio (χ^2^/df = 2.15) was below the recommended threshold of 3, indicating acceptable model fit. The Root Mean Square Error of Approximation (RMSEA = 0.056) was within the acceptable range (< 0.08). Furthermore, the Comparative Fit Index (CFI = 0.962) and the Goodness-of-Fit Index (GFI = 0.954) both exceeded the conventional cutoff of 0.95, suggesting excellent model fit. The Standardized Root Mean Square Residual (SRMR = 0.041) was also below the recommended maximum of 0.08, further confirming model adequacy.

Collectively, these indices demonstrate that the hypothesized model provides a robust and wellfitting representation of the data, consistent with established guidelines (Hu and Bentler, 1999; Jöreskog and Sörbom, 1993; Kline, 2016). All statistical analyses were conducted using AMOS 23 for path analysis, and SPSS 27 for descriptive statistics, assumption testing, and preliminary correlation analyses.

## 3 Results

The study’s findings are presented as follows: first, the relationship between the variables that make up the hypothesis of the study and descriptive statistical values are explained (see Table 2). This is followed by findings showing whether the hypotheses in the research model were accepted or not (see Tables 3–5).

**Table 3:** Results of path analysis.

Predictive variables	Outcome variables										
	Motor creativity (Y)					Motivation (M)					
	B	β	SE	C.R	*p*	B	β	SE	C.R	*p*	Hypotheses
Risky play (X)	0.328	0.320	0.024	54.839	0.000	0.903	0.953	0.037	41.815	0.000	H_1_/H_2_
Motivation (M)	0.435	0.478	0.054	58.601	0.000						H_3_

B = Unstandardized Estimates; β = Standardized Estimates; CR, Critical Ratio.

**Table 4:** Mediation effect path analysis.

Outcome variable	Predictive variable					
Motor creativity (Y)	Risky play (X)					
	B	β	95% CI		*p*	Hypotheses
			Lower	Upper		
Standardized Total Effect	0.816	0.465	0.774	0.845	0.000	H_3a_
Standardized Indirect Effect	0.018	0.013	0.436	0.486	0.000	

B = unstandardized estimates; β = standardized estimates; CI, Confidence Interval.

**Table 5:** Moderator variable effect model path analysis results.

Predictive variable	➔	Outcome variable	Gender (W)										Hypotheses
			Girl (path a)					Boy (path b)					
			B	β	SE	CR	*p*	B	β	SE	CR	*p*	
Risky Play (X)	➔	Motivation (M)	1.295	0.902	0.037	35.470	0.000	1.39	0.950	0.021	67.680	0.000	H_4a_
Z (path b)			2.257					-					
Motivation (M) R^2^			0.813					0.903					
Risky Play (X)	➔	Motor Creativity (Y)	1.415	0.357	0.019	73.875	0.000	0.973	0.383	0.042	23.319	0.000	H_4b_
Z (path b)			9.087					-					
Motivation (M)	➔	Motor Creativity (Y)	1.391	0.514	0.018	75.377	0.000	0.856	0.591	0.106	8.074	000	H_4c_
Z (path b)			4.879					-					
Motor Creativity (Y) R^2^			0.722					0.695					

B, Unstandardized Estimates; β, Standardized Estimates; CI, Confidence Interval.

Table 2 presents the relationships among parental perception toward risky play, mastery motivation, and motor creativity, along with the means, standard deviations, skewness, and kurtosis values for each variable. Additionally, Pearson’s r correlation coefficients are shown for the entire sample and by gender (female and male). In the full sample (*N* = 367), significant positive relationships are observed between risky play, motivation, and motor creativity (all *p* < 0.01). The strongest relationship is found between risky play and motor creativity (r = 0.790), followed by the relationship between risky play and motivation (r = 0.736). Descriptive statistics indicated that participants’ mean scores were 77.24 (SD = 9.51) for risky play, 88.72 (SD = 14.47) for motivation, and 261.26 (SD = 31.87) for motor creativity. Skewness and kurtosis values indicate that the variables follow a normal distribution. When examining the groups by gender, girls (N = 182) show slightly higher mean scores in motor creativity (265.05) compared to boys (257.54). However, the correlation patterns are similar for both groups. For example, the correlation between risky play and motivation is slightly higher in boys (r = 0.753) than in girls (r = 0.719). Skewness and kurtosis values indicate that the data for both groups follow a normal distribution.

Path analysis was applied to test the hypotheses established for parental perceptions toward risky play, mastery motivation, and motor creativity which are the variables of the study. H_1_, H_2_, and H_3_ analysis results are given in Table 3.

According to Table 3, a significant positive association is observed between parental perceptions toward risky play and motor creativity (*β* = 0.320; CR = 54.839; *p* < 0.01), providing support for *H_1_*. Additionally, parental attitudes toward risky play and mastery motivation are significantly and positively associated (*β* = 0.953; CR = 41.815; *p* < 0.01), supporting *H_2_*. Furthermore, mastery motivation is positively associated with motor creativity (*β* = 0.478; CR = 58.601; *p* < 0.01), supporting *H_3_*. These findings indicate that children whose parents hold more supportive perception toward risky play tend to exhibit higher levels of mastery motivation and motor creativity.

**Figure 2:**
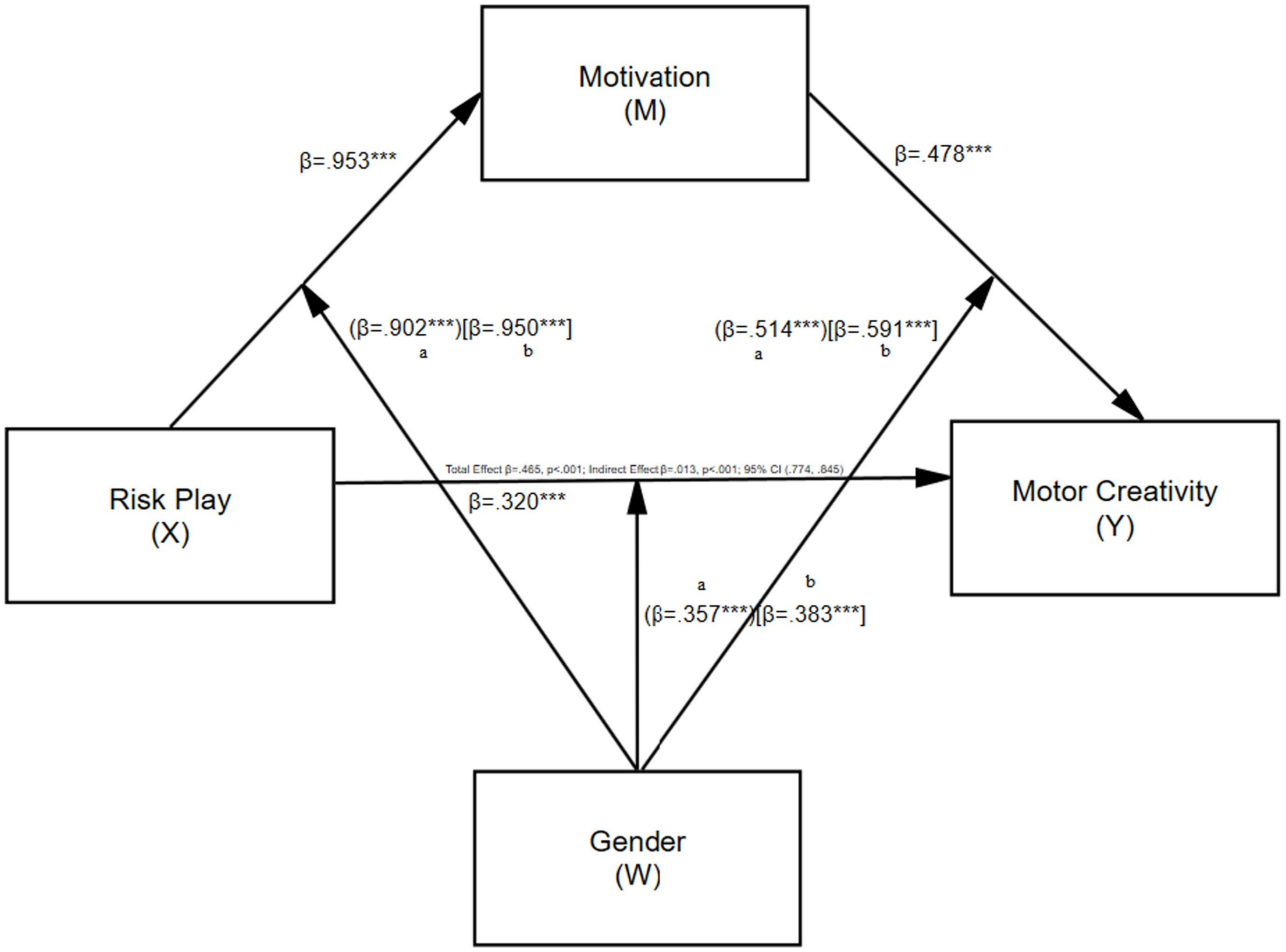
Standardized estimate values of the model as a result of path analysis.

The explanatory power of the model (see Figure 2) shows that mastery motivation accounts for 90% of the variance (R^2^ = 0.90), indicating that mastery motivation is a very strong predictor within the model. Similarly, motor creativity accounts for 72% of the variance (R^2^ = 0.72), suggesting that the model explains a substantial proportion of individual differences in children’s motor creativity. To further test the mechanism underlying these relationships, a mediation analysis was conducted to examine whether mastery motivation mediates the association between parental perception toward risky play and motor creativity (see Figure 2; Table 4).

According to the results presented in Table 4, the relationship between parental perception toward risky play (X) and motor creativity (Y) was examined through standardized total and indirect effects, with the bootstrap results providing further confirmation. The standardized total effect indicates a significant positive association between parental perception toward risky play and motor creativity (*β* = 0.465; 95% CI [0.774, 0.845]; *p* < 0.01), supporting *H_3a_*. The bootstrap confidence interval (CI) confirms that this association is robust and statistically significant.

The standardized indirect effect (β = 0.013; 95% CI [0.436, 0.486]; p < 0.01) suggests that mastery motivation serves as a partial mediator in this relationship. This finding indicates that while parental attitudes toward risky play are directly related to motor creativity, part of this association is explained through mastery motivation. The bootstrap analysis further supports the significance of this indirect effect.

When Table 4 is examined, it is observed that the coefficient of mastery motivation (M) in the simple effect model decreases in the mediation model where parental perception toward risky play (X) and motor creativity (Y) are both included. Since the confidence interval (CI) for the mediating effect does not contain zero, it can be concluded that mastery motivation significantly mediates the association between parental perception toward risky play and motor creativity. Hence, *H_3a_* is supported. When comparing the results of the standardized indirect effect test (representing the total effect) and the mediation model (representing the simple effect model), the strength of the association between parental perception toward risky play and motor creativity decreases, while remaining statistically significant. This suggests that mastery motivation plays a partial mediating role in this relationship.

Following this analysis, a moderation effect analysis was conducted to determine whether gender moderates the relationships between parental perception toward risky play, mastery motivation, and motor creativity (H_4a_, H_4b_, H_4c_). The findings of these analyses are presented in Table 5.

Table 5 presents the analysis of the relationship between parental perception toward risky play, mastery motivation, and motor creativity, considering the moderating role of gender. The findings indicate significant differences between girls and boys.

The association between parental perception toward risky play and mastery motivation is statistically significant for both girls (*β* = 0.902; CR = 35.470; *p* < 0.01) and boys (*β* = 0.950; CR = 67.680; *p* < 0.01). However, the Z value (Z = 2.257; Z > 1.96) suggests that this association is stronger for boys. Similarly, the association between parental perception toward risky play and motor creativity is significant for both girls (*β* = 0.357; CR = 73.875; *p* < 0.01) and boys (*β* = 0.383; CR = 23.319; *p* < 0.01), with the Z value (*Z* = 9.087) indicating a stronger relationship for boys. Additionally, the relationship between mastery motivation and motor creativity is significant for both girls (β = 0.514; CR = 75.377; *p* < 0.01) and boys (*β* = 0.591; CR = 8.074; *p* < 0.01). The Z value (Z = 4.879; Z > 1.96) again suggests that this association is stronger for boys.

Regarding the explanatory power of the model, mastery motivation accounts for 81% of the variance in girls (R^2^ = 0.813) and 90% in boys (R^2^ = 0.903). Similarly, motor creativity accounts for 72% of the variance in girls (R^2^ = 0.722) and 69% in boys (R^2^ = 0.695). These findings suggest that the relationships among parental perception toward risky play, mastery motivation, and motor creativity differ by gender. The stronger associations observed in boys imply that gender differences should be considered when developing interventions or educational programs aimed at fostering motor creativity through risky play-related parental attitudes.

## 4 Discussion

This study aimed to examine the role of parental perceptions of risky play in early childhood motor creativity and to determine the mediating effect of motivation and the moderating effect of gender. The findings support the association between parental perceptions of risky play and motor creativity, confirm that motivation mediates the relationship between parental perceptions of risky play and motor creativity, and show that gender moderates the relationships among parental perceptions of risky play, motivation, and motor creativity.

Our first hypothesis received empirical support, confirming the association between parental perceptions of risky play and motor creativity (H_1_). Children whose parents exhibit more supportive perceptions toward risky play tend to display higher levels of motor creativity. Because parental perceptions shape children’s access to diverse play experiences, these perceptions may either facilitate or restrict opportunities for creative motor exploration. Recent evidence also supports this interpretation, showing that parents’ perceptions of risk in play are closely related to children’s opportunities to engage in outdoor and risky play, as parents and caregivers often act as gatekeepers who either enable or limit children’s access to challenging play experiences (Ryan et al., 2024; Sturges et al., 2023). Prior studies have shown that when educators and caregivers recognize the developmental benefits of risky play, children demonstrate greater fluency and originality in their movement patterns (Karaca et al., 2024). This suggests that beyond children’s own dispositions, adult perceptions and support systems play a key role in shaping opportunities for creative movement.

At the same time, this also confirms our hypothesis that “Gender moderates the effect of parental perceptions of risky play on motor creativity, with boys scoring higher than girls (H_4b_).” Observational studies indicate that while boys tend to prefer more vigorous play, girls more often engage in structured forms (e.g., opting for dramatic play centers rather than hopping and jumping); boys participate directly in risky play, whereas girls frequently take observational or supervisory roles pointing, commenting, or watching rather than physically engaging (Gleave, 2008; Reis et al., 2021). Furthermore, a study by Sando et al. shows that boys are more willing than girls to participate in physically demanding and risk-taking activities (Sando et al., 2025). This aligns with research showing that unstructured, physically engaging play environments contribute to creative problem-solving and adaptive movement patterns (Boyer, 2006: Stephenson, 2003). When children are presented with a problem in unstructured play environments, both risky play and motor creativity can be supported.

These findings contribute to ongoing debates about the role of risk-taking opportunities in child development. Recent research also highlights that risk in children’s play is often shaped by policy and institutional discourses, where risk-averse perspectives may limit opportunities for physically active play despite its developmental benefits (Jerebine et al., 2025). Further reinforces this perspective by emphasizing that risk in play should not be viewed solely as a negative factor, but rather as a necessary component of learning environments that support children’s physical, cognitive, and socio-emotional development through a balanced evaluation of both benefits and potential harms (Eager et al., 2025). Research has shown that overprotective parenting and increasingly stringent safety regulations in play environments reduce children’s engagement in risky play (Brussoni et al., 2012; Little and Eager, 2010). However, such restrictions may inadvertently limit opportunities to develop foundational motor skills. Prior work indicates that parents who have greater intolerance of uncertainty and adopt more overprotective styles are more likely to restrict children’s outdoor and exploratory play (Oliver et al., 2022). Studies emphasize that physically challenging play experiences are associated with improved coordination (Schweizer, 2009), spatial awareness (Sandseter, 2010), and risk assessment skills (Little and Wyver, 2008; Thomas and Harding, 2011). Despite these benefits, some parents and educators hesitate to encourage risky play due to concerns about injury (Ball et al., 2012; Malone, 2007).

Our second hypothesis that parental perceptions of risky play directly and positively affect motivation (H_2_) was also supported. The pleasure or excitement of play constitutes a fundamental motivational basis for children (Smith, 1982). Motivation is especially potent in risky play (Sandseter, 2009b). Recent evidence suggests that play-based experiences support creativity through self-regulation processes, which enable children to sustain engagement, manage behavior, and generate creative responses during play activities (Ghanamah, 2026). Risky play is an enjoyable form of exercise that includes intervals of cardiovascular activity which stimulate healthy processing and learning to manage moderate stress, thereby providing motivation (Poulson and Ziviani, 2004). Sutton-Smith (1997) argues that a child’s personal reason for play is an intrinsic motivation to experience positive emotional stages such as arousal, excitement, fun, joy, ecstasy, mastery, and competence. In early childhood, the desire to continuously increase physical challenges along with the wish to impress peers, curiosity, and the urge to meet challenges can serve as motivation for risky play and can be interpreted as the learning dimension of motivated engagement in risky play. Parental support is particularly necessary for children’s participation in risky play. In early childhood, parents play a primary role in providing or permitting environments where risk can be taken. In this regard, Hyson and Bollin (1990) suggested that as parental supervision decreases with children’s age, children’s internal evaluations of risk may increasingly influence their major risk-taking decisions. According to Maccoby and Martin (1983), parental responsiveness, being sensitive and supportive to a child’s particular needs and desires is important for personality development; similarly, Leung and Kwan (1998) found that parents who support children’s emotions and needs and adopt democratic parenting attitudes tend to have highly motivated children.

Moreover, gender is known to influence parental perceptions of risky play. Our hypothesis “Gender moderates the relationship between parental perceptions of risky play and motivation, with boys scoring higher than girls (H_4a_)”—is supported by our findings. According to the results, parental perceptions of risky play are statistically significant for motivation among both girls and boys; however, in these relationships, boys’ scores are higher than girls’, as reported in prior studies. These results are aligned with earlier research showing that boys are more inclined than girls to engage in physically riskier play, which may in turn enhance their motivation and motor creativity (Little, 2015; Little and Wyver, 2008; Play Wales, 2023; Sandseter and Kennair, 2011; Tovey, 2010). This pattern is consistent with recent findings indicating that boys tend to engage more frequently in physically active and risk-related play behaviors, while girls may participate less often in such activities, reflecting differences in play preferences and opportunities (Sando et al., 2025). Therefore, parents should adopt supportive and encouraging attitudes not only toward boys but also toward girls when it comes to risky play. At the same time, it is emphasized that rather than eliminating all risk in outdoor environments, parents should encourage risky play within a “controlled and supportive” context (Ofsted, 2006). The literature also includes studies identifying motivation as a key factor in children’s play/risky play (Meluso et al., 2012; Meşe and Dursun, 2018; Sarı and Altun, 2016).

Our third hypothesis—that motivation directly and positively affects motor creativity (H_3_)—was supported. Consistent with the hypothesis, there is a strong relationship between motivation and motor creativity. Motivation—viewed as an important psychological mechanism that drives individuals to initiate, sustain, and persist in activities (Ryan and Deci, 2000; Vasilopoulos and Dumontheil, 2024) influences children’s willingness to engage in challenging activities and to try new movement patterns (Carlton and Winsler, 1998). By arousing children through the excitement experienced during play, motivation supports the manipulation of ideas, the generation of new ideas, and the exploration of various movement solutions, thereby enhancing motor creativity (Fung and Chung, 2022; Murayama and Elliot, 2009; Topçu, 2015). This perspective is further supported by recent theoretical work highlighting that motor activity and creativity are interconnected through shared cognitive and exploratory processes, contributing to holistic child development (Pesce et al., 2025). Recent research further indicates that motor creativity is not only an outcome of such engagement but also a process that supports adaptive learning and flexible problem-solving in children, reinforcing its role as a key mechanism in development (Ghanamah and Pesce, 2026). Although there are few direct studies on the relationship between motor creativity and motivation, activities that strongly influence children’s motivation—such as sport and physical activity, music education and dance, and play—have been shown to support motor creativity (Coppola et al., 2024; Karaca and Aral, 2017; Khorkova et al., 2024; Pamuk et al., 2022; Panagiotaki et al., 2023; Richard et al., 2018; Şenol and Karaca, 2025; Thomaidou et al., 2021; Tocci et al., 2022).

The findings also confirm our hypothesis that motivation mediates the significant relationship between parental perceptions of risky play and motor creativity (H_3a_). Accordingly, when parents encourage children to engage in risky play, children develop a stronger motivational drive, which is associated with increased motor creativity. Motivation defined as persistence in overcoming challenges and developing new skills plays a fundamental role in competence development (Morgan et al., 1990; Saracho, 2019; Shonkoff and Phillips, 2000). These results are consistent with effectance motivation theory, which posits that individuals are driven to interact with and master their environments (White, 1959). White (1959) emphasized that effectance motivation is especially in dynamic and uncertain contexts, the core characteristics of risky play facilitate creativity and problem-solving. Research has further shown that motivation is shaped by prior experiences of success and failure, the availability of appropriately challenging tasks, and external reinforcement from caregivers (Gilmore and Cuskelly, 2025). In addition, studies indicate that highly motivated children persist longer on problem-solving tasks and explore more alternative solutions, leading to greater creative flexibility (Beghetto and Kaufman, 2016; Carlton and Winsler, 1998; Renshaw et al., 2009; Runco and Acar, 2012).

In the context of risky play, when children successfully overcome physical challenges, they may experience a sense of accomplishment that strengthens their motivation to engage in exploratory movement activities. However, given that children in early childhood are young and dependent on their parents for decision-making, parental support is required for play environments and materials. Therefore, it is known that gender also influences parental perceptions when determining play contexts such as risky play and motor creativity.

Our final hypothesis, that gender moderates the relationship between motivation and motor creativity (H_4c_) is supported. Children in early childhood are known to possess a natural inclination and enthusiasm for engaging in movement activities and physically active play, as well as vast creative potential and motivation for problem-solving. According to our findings, both boys’ and girls’ motivation levels positively influence their motor creativity skills; however, mean scores indicate that boys score higher than girls. Studies on gender differences underscore socialization as the most important factor, highlighting the influence of adults’ gendered attitudes (Brussoni et al., 2015; Gleave, 2008; Little, 2015). Cultural and societal expectations often encourage boys to participate in physically intense, risk-oriented activities, while girls may be directed toward structured or cooperative play (Gleave, 2008; Play Wales, 2023). Such norms shape the extent to which children experience the benefits of risky play and may lead to stronger effects for boys. Moreover, adult supervision and feedback appear to reinforce these gender differences. As noted in prior research, both children and adults often produce supervisory comments that amplify fear and actively discourage girls from participating in risky play (Reis et al., 2021). Conversely, the relatively weaker relationship observed among girls suggests that additional factors, such as social cooperation, environmental constraints, or different parental expectations may influence their participation in risky play and its impact on motor creativity (Brussoni et al., 2012; Little and Wyver, 2008).

Interestingly, research indicates that girls’ lower participation in risky play does not necessarily reflect a lack of interest, but rather fewer opportunities and less social encouragement (Little, 2015). Recent research also highlights that access to play opportunities is strongly shaped by environmental and social structures, suggesting that limitations in children’s engagement with challenging play are often the result of contextual constraints rather than individual preferences, particularly for girls (Eager et al., 2025). Parental safety concerns and societal expectations regarding “appropriate” play behaviors for girls may contribute to these disparities (Gull et al., 2018). Additionally, restrictive parenting styles and overprotective tendencies are associated with reduced opportunities for girls to engage in physically challenging play, potentially limiting their exposure to activities that promote motor creativity. Among boys, motivation, defined as the persistent effort to overcome challenges and develop competence, has been shown to play a crucial role in the exploration of motor creativity (Morgan et al., 1990; Saracho, 2019). Because boys are often encouraged to persist in physically demanding tasks, they tend to exhibit a stronger motivational drive in risky play, which in turn supports the development of creative motor solutions (Gleave, 2008; Karaca et al., 2024). These findings also invite reflection on contemporary movement-based educational approaches that aim to enhance cognitive functions while managing physical risk within structured learning environments. For example, programs such as Brain Breaks (Jenson, 2000) and mini-eduball interventions integrate brief bouts of physical activity into academic routines, thereby linking motor engagement with cognitive stimulation and offering a structured alternative to traditionally unstructured risky play. In parallel, evidence from school-based interventions, including active lunch breaks and recess periods, demonstrates that even within structured school contexts, opportunities for physically active play are associated with increased engagement in exploratory and risk-taking behaviors, alongside positive developmental outcomes (Ridgers et al., 2011). Furthermore, research on purposefully designed playgrounds suggests that environments offering calibrated or ‘managed’ risk can effectively foster children’s creativity, problem-solving, and adaptive motor behaviors (Brussoni et al., 2017).

Ultimately, these insights underscore that the developmental benefits associated with risky play are not confined to unstructured outdoor contexts; rather, they can be intentionally cultivated through thoughtfully designed educational programs and physical environments that strategically balance safety with challenge.

## 5 Conclusion and recommendations

This study provides empirical support for the role of parental perceptions of risky play in promoting motor creativity among preschool children, examining motivation as a mediating factor and gender as a moderating factor. The findings indicate that children whose parents adopt more supportive perceptions toward risky play exhibit higher levels of motivation, which in turn contributes to greater motor creativity. Moreover, the relationships among these variables differ by gender, with boys showing stronger associations than girls between parental perceptions of risky play, motivation, and motor creativity. These results underscore the developmental value of risky play and motivation and highlight the importance of family support and social norms in shaping children’s creative motor behaviors. By drawing attention to the conceptualization of motor creativity, an area of particular importance in early childhood development and its connections with other developmental domains, the findings offer meaningful implications for classroom practice. The study also emphasizes the need for a balanced approach to risky play in early childhood, ensuring that children have opportunities to explore, experiment, and take calculated risks in ways that enhance their motivation and creativity, while foregrounding the role of parental perceptions in this process.

Despite these contributions, the study has several limitations. First, its cross-sectional design precludes causal inferences regarding the relationships among parental perceptions, motivation, and motor creativity. Longitudinal research is needed to examine how these relationships evolve over time and whether changes in parental perceptions influence children’s motivation and the development of their creative motor skills. In addition, relying on parent reports to assess perceptions of risky play may introduce subjective bias, as parental perceptions may not always align with children’s actual engagement in risky play. Future research should incorporate direct behavioral observations or educator ratings to enhance measurement accuracy. Subsequent work should consider these variables to achieve a more comprehensive understanding of the mechanisms shaping children’s motor creativity. Future studies should also explore different types of risky play and their specific effects on motivation and creativity. Longitudinal designs could illuminate the long-term benefits of early engagement in risky play, particularly in relation to problem-solving, flexibility, and adaptive movement skills. Cross-cultural research could further investigate how societal perceptions of risk-taking influence children’s play behaviors and developmental outcomes. Finally, evaluating interventions that promote risk-positive play in early childhood education settings and assessing their effectiveness in fostering creativity across diverse populations would strengthen the practical implications of this research.

## Data Availability

The raw data supporting the conclusions of this article will be made available by the authors, without undue reservation.
